# Conductivity and Pseudocapacitance Optimization of Bimetallic Antimony–Indium Sulfide Anodes for Sodium‐Ion Batteries with Favorable Kinetics

**DOI:** 10.1002/advs.201800613

**Published:** 2018-07-26

**Authors:** Yongxin Huang, Ziheng Wang, Ying Jiang, Shuaijie Li, Min Wang, Yusheng Ye, Feng Wu, Man Xie, Li Li, Renjie Chen

**Affiliations:** ^1^ Beijing Institute of Technology School of Materials Science and Engineering Beijing 100081 China

**Keywords:** anodes, heterostructures, Sb_2_S_3_, sodium‐ion batteries

## Abstract

Metal sulfides show promise for use in alkali‐ion batteries because of their high theoretical capacities. However, their poor cycling stability and rate performance hinder their further development. To avoid these issues, In_2_S_3_ into Sb_2_S_3_ is introduced to improve its electrochemical properties by optimizing its crystal structure and sodium storage mechanism. A heterostructure composed of In_2_S_3_ and Sb_2_S_3_ shows a unique morphology of formicary microspheres, which provide abundant channels for fast transfer of sodium ions, large surface area for a high pseudocapacitance effect, and enough voids to relieve volume expansion. A sodium‐ion battery containing the bimetallic sulfide anode exhibits a high reversible capacity of 400 mA h g^−1^ and long cycle life of about 1000 cycles. Similarly, a high capacity of ≈610 mA h g^−1^ is achieved for a lithium‐ion battery containing the anode. During sodiation/desodiation, the synergistic effect of In_2_S_3_ and Sb_2_S_3_ enhances electronic conductivity and supports the host structure, preventing collapse. The cycling performance and rate performance of the In_2_S_3_–Sb_2_S_3_ anode are further improved by wrapping the electrode with carbon nanotubes. Even at a high current density of 3.2 A g^−1^, this carbon composite structure still shows a capacity of about 355 mA h g^−1^.

## 1 Introduction

Sodium‐ion batteries (SIBs) are considered as potential alternatives to lithium‐ion batteries (LIBs), and in past years, development of the cathode, anode, and electrolyte of SIBs has progressed rapidly.^1^, ^2^, ^3^, ^4^ For instance, layered transition metal oxides,^5^ polyanionic compounds,^6^ and Prussian blue and its analogues^7^ have received much attention for use in SIBs because of their stable electrochemical properties and easy synthesis. Moreover, various electrolytes^8^ and additives^9^ have been developed to provide safe SIBs with high energy density. In efforts to further improve the energy storage efficiency and safety of SIBs, metal sulfides have shown promise as anodes because of their high specific capacity and working voltage.^10^, ^11^, ^12^ Among metal sulfides, Sb_2_S_3_ exhibits an ultrahigh theoretical capacity of 946 mA h g^−1^, corresponding to the transfer of 12 mol of electrons and sodium ions (Na^+^).^13^ Nevertheless, this material has many shortcomings that must be solved before it can be used in practical applications. First, Na^+^ insertion causes huge volume expansion, which pulverizes the electrode materials.^14^ In particular, the alloying reaction between Na^+^ and metallic antimony to form Na_3_Sb may cause violent growth to ≈400% of the original volume.^15^, ^16^ Second, the insertion/extraction rate of Na^+^ is limited by its sluggish diffusion in bulk Sb_2_S_3_ and the small contact area between the electrolyte and bulk electrode.^10^ Thus, actual batteries with pure bulk Sb_2_S_3_ anodes are likely to have low power density and poor cycling stability. Third, the low electronic conductivity of Sb_2_S_3_ of below 1 × 10^−5^ S cm^−1^ hinders fast electron transfer in its electrodes.^17^

Various modifications have been used to try to overcome the sluggish kinetics and large volume change associated with Na^+^ storage in Sb_2_S_3_ electrodes. One effective way to enhance the reaction kinetics of Sb_2_S_3_ electrodes is to design novel nanostructures with a short diffusion distance for Na^+^ and large interfaces between the electrolyte and electrode.^18^ For instance, a flower‐like Sb_2_S_3_ anode self‐assembled from nanosheets showed enhanced electrochemical properties compared with those of bulk Sb_2_S_3_, which was ascribed to the effective volume buffering and Na^+^ transport.^19^ Notably, the sodiation kinetics of an Sb_2_S_3_ electrode is subject to the 1D van der Waals force.^20^ Because of this behavior, 1D rod‐like Sb_2_S_3_, with long‐range lattice order and the nanosize effect, displayed good charge‐transfer kinetics and high specific surface area.^21^ High‐performance Sb_2_S_3_ electrodes can also be produced by combination with carbon materials. For example, in situ growth of Sb_2_S_3_ on multiwalled carbon nanotubes (MCNTs) produced materials with high capacity and good stability because of the 3D porous networks of the MCNTs.^22^ Zhao and Manthiram^23^ used Sb_2_S_3_ embedded in graphite as a long‐life, high‐rate anode for SIBs. Graphite not only acts as a conductive matrix, but also provides a buffer between Sb_2_S_3_ nanoparticles, relieving volume expansion. Xiong et al. showed that sulfur‐doped graphene sheets can be tightly connected to Sb_2_S_3_, providing high electronic conductivity and protecting the intermediate products of Sb and Na_3_Sb.^24^

Based on previous reports on alloying anodes, a promising strategy to improve both structural stability and electronic conductivity is constructing *M*–(Sn, Sb, Ge, etc.) intermetallics, where *M* is an electrochemically inactive component in the system.^25^, ^26^, ^27^ After the first cycle, the intermetallics convert to a composite of *M* and active alloys. *M* not only acts as a buffer layer between the Na–alloy phases, but also forms a conductive network to allow rapid electron transport in the electrode. Therefore, *M* (Al, Cu, Mo, Bi, etc.) can be introduced into Sb_2_S_3_ electrodes to improve their performance.^28^, ^29^ An electrode composed of a solid solution of Bi_2_S_3_ and Sb_2_S_3_ exhibited high capacity retention of 79% after 200 cycles, which can be ascribed to the synergistic effect of these two components.^30^ Recently, it was shown that the formation of Bi–Sb alloy during the cycling process can promote the reversibility of Na^+^ storage, which may provide a new strategy to design bimetallic sulfides.^31^ In addition to solid solution structure, many kinds of heterostructures have been developed to improve reaction kinetics through the effect of a built‐in electric field.^32^ For instance, an SnS/SnO_2_ heterostructure delivered outstanding durability at high current density because of its enhanced electronic transport properties compared with those of SnO_2_ alone.^33^

In this study, we form a heterostructure of In_2_S_3_ and Sb_2_S_3_ (denoted as I–S) that displays synergistically enhanced electrochemical properties by a simple synthetic route. I–S contains abundant pores and a rather high specific surface area, which not only improve the Na^+^ storage dynamics of the material via a strong pseudocapacitance effect, but also provide space to allow volume expansion during the storage process. The indium(III) ions (In^3+^) introduced to Sb_2_S_3_ influence the final structure of I–S because they induce crystal growth. Moreover, the low reversibility of the conversion reaction between In_2_S_3_ and the electrochemically inert In lead to the formation of an In buffer layer, which suppresses volume expansion. To build a 3D conductive network, we prepare a composite of MCNTs and I–S particles (denoted as I–S@MCNTs).

## 2 Results and Discussion

**Figure 1:**
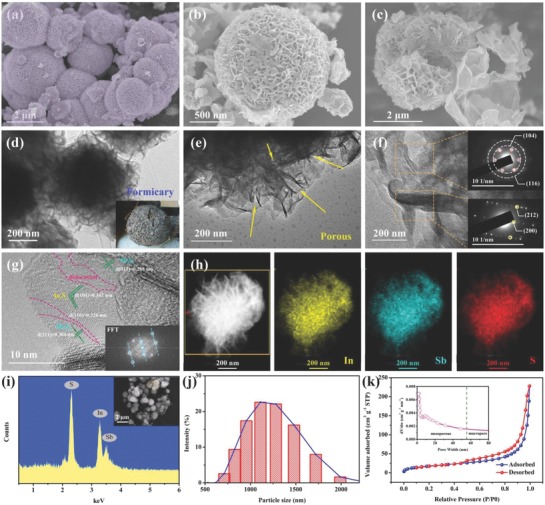
a–c) SEM images and d–f) TEM images of formicary‐like I–S microspheres with different magnifications and perspectives; the insets in panels f are the SAED patterns of local areas. g) HRTEM image and corresponding fast Fourier transform (FFT) pattern of I–S sample. h) EDX mapping images of In, Sb, and S elements with corresponding HAADF image. i) EDS pattern of the I–S sample with corresponding SEM image. j) Particle size distribution histogram of I–S sample. k) N_2_ adsorption–desorption isotherms and pore size analysis of I–S sample.

The I–S sample was investigated by scanning electron microscopy (SEM). **Figure** **1**a shows that I–S consisted of porous microspheres. Closer observation (Figure 1b) revealed that the hierarchical spheres were assembled from crumpled nanosheets with thicknesses of 5–8 nm, which provided open channels for sufficient electrolyte infiltration and fast Na^+^ transfer. A cross‐sectional image of the I–S microspheres (Figure 1c) illustrated that they contained many interspaces in their centers, which can tolerate volume expansion during Na^+^ storage. The detailed morphology and crystal structure of I–S were observed by transmission electron microscopy (TEM) and high‐resolution transmission electron microscopy (HRTEM). Figure 1d shows a complete I–S sphere, which has a porous structure and large specific surface area, which may originate from its formicary structure, as shown in the inset. The enlarged image in Figure 1e reveals that the nanochannels are connected, which should facilitate the transport of Na^+^. Selected‐area electron diffraction (SAED) patterns obtained from different regions of a particle (Figure 1f) contained characteristic signals from the (104), (116), (212), and (200) crystal planes of In_2_S_3_ and Sb_2_S_3_.^21^, ^34^ As shown in an HRTEM image and corresponding fast Fourier transform (FFT) image (Figure 1g), each monolayer I–S sheet is composed of Sb_2_S_3_, with signals observed from the (013) and (211) planes, as well as In_2_S_3_, with signals from the (104) and (110) planes detected, which are connected by dislocation areas. The FFT image further corroborated the hierarchical structure of I–S consisting of different crystal systems. These results show that the Sb_2_S_3_ and In_2_S_3_ phases were tightly bound by chemical bonds.^24^ Furthermore, the different crystal planes of these two compounds were interconnected by mesophase areas to form a uniform heterostructure, as shown in Figure S1 of the Supporting Information.

The center dark field image of I–S in Figure 1h shows a distinct net‐like structure with well‐defined boundaries, revealing the high crystallinity of I–S sample, which was accomplished without energy‐intensive calcination. As shown in the corresponding energy‐dispersive X‐ray (EDX) maps, the distributions of In, Sb, and S indicated that uniform I–S nanoparticles containing strong chemical bonds were formed, and the presence of voids verified the presence of abundant interior pores. The exact contents of In, Sb, and S in the I–S sample were confirmed by energy‐dispersive spectroscopy (EDS) (Figure 1i) and inductively coupled plasma atomic emission spectrometry (ICP‐AES) (Table S1, Supporting Information). The composition of I–S can be described as In_0.63_Sb_1.37_S_3_. In addition, composite formation did not change the elemental contents of I–S (In_0.66_Sb_1.34_S_3_@MCNTs). Note that the spherical I–S microparticles (Figure 1j) provide high volumetric energy density because they are tightly packed. As shown in the N_2_ adsorption–desorption isotherms in Figure 1k, the I–S sample had a Brunauer–Emmett–Teller (BET) surface area of 66.87 m^2^ g^−1^, which is markedly improved compared with that of bulk Sb_2_S_3_ because of its novel formicary structure. Meanwhile, the average pore size of I–S was less than 15 nm and its total pore volume was 0.353 cm^3^ g^−1^ (i.e., porosity of ≈74%), indicating a mesoporous structure. Further analysis of pore size revealed that the I–S particles had both microporous and mesoporous structures. Thus, the electrolyte can fully contact the surface and interspace of I–S, which shortens the propagation distance of Na^+^.

The low electron transmission rate of common metal sulfides may cause sluggish reaction kinetics. To avoid this problem, we composited an I–S sample with ultrafine MCNTs by in situ growth. As shown in Figure S2a of the Supporting Information, the I–S@MCNT composites contained tightly connected I–S and MCNTs, forming a 3D conductive network that can raise electron and ion transmission rates and prevent particle agglomeration. Meanwhile, the uniform distributions of In, Sb, and S in the nanoparticles reveal that composite formation did not change the original material properties of I–S (Figure S2b, Supporting Information).

**Scheme 1:**
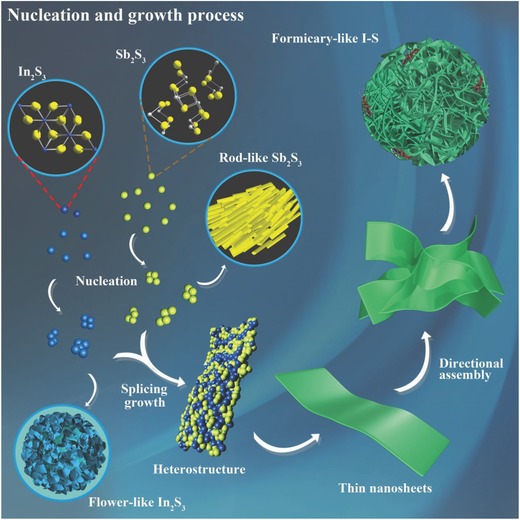
Schematic illustration of the self‐assembly routes of rod‐like Sb_2_S_3_, flower‐like In_2_S_3_, and formicary‐like I–S particles.

Based on complementary experiments and previous reports,^21^, ^34^, ^35^ the formation of antimony and indium sulfides with different morphologies is predicted to occur by the process shown in **Scheme** **1**, which has six steps. At the beginning of the liquid phase reaction, the metal cations (Sb^3+^ and In^3+^) and sulfur anions (S^2−^) combine to form In_2_S_3_ and Sb_2_S_3_ nuclei, respectively. As the temperature and pressure in the stainless steel autoclave increase, the crystal nuclei gradually grow in a certain direction to form nanosheets along the facets with low formation energy, which is guided by the ethanediol solvent and interaction between In_2_S_3_ and Sb_2_S_3_ particles.^36^ Likewise, rod‐like Sb_2_S_3_ was obtained in a solution without In^3+^ (Figure S3a, Supporting Information), whereas flower‐like In_2_S_3_ was produced in a solution without Sb^3+^ under the same conditions (Figure S3b, Supporting Information). When the reaction was maintained at high temperature for long enough, the folded nanosheets including Sb_2_S_3_ and In_2_S_3_ phases assembled by electrostatic forces into porous spheres with large specific surface areas: these are the formicary structures. To improve the electron transfer between isolated I–S particles, we composited them with MCNTs, which did not change their porous spherical morphology.

**Figure 2:**
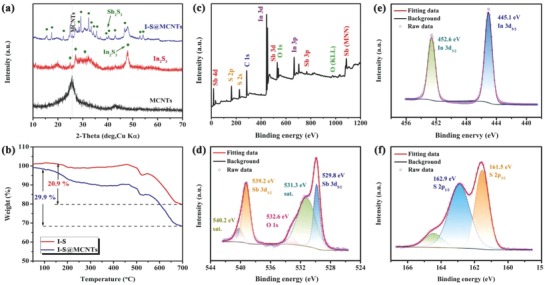
a) XRD patterns of MCNTs, I–S, and I–S@MCNTs samples. b) TG curves of ISS and ISS@MCNTs samples. c) The XPS survey spectrum and d) Sb 3d, e) In 3d, and f) S 2p high‐resolution XPS spectra of the I–S@MCNTs composite.

The X‐ray diffraction (XRD) patterns of I–S and I–S@MCNTs (**Figure** **2**a) show peaks from both In_2_S_3_ with a rhombohedral structure and Sb_2_S_3_ with an orthorhombic structure, indicating a composite phase of In_2_S_3_ and Sb_2_S_3_. The peaks at 2θ of 29.3°, 32.3°, and 35.5° can be well indexed to the (211), (221), and (240) crystal planes of Sb_2_S_3_ (Figure S4, Supporting Information), respectively.^21^ Meanwhile, the peaks at 26.6°, 28.7°, and 33.3° correspond to the (110), (101), and (102) crystal planes of In_2_S_3_, respectively.^37^ It can be inferred that the Sb_2_S_3_ phase is in the *Pbnm* space group and that the In_2_S_3_ phase is in the *P3m1* space group. I–S was successfully composited with MCNTs, as verified by the diffraction peak at 25.6° in the pattern obtained for in the I–S@MCNTs that corresponds to the (002) crystal plane of the MCNTs. The coexistence of Sb_2_S_3_ and In_2_S_3_ in the I–S@MCNTs was further confirmed by Raman spectroscopy (Figure S5, Supporting Information). The peaks observed at 266 and 312 cm^−1^ are consistent with vibrations of the homopolar bonds of Sb–Sb and In–In, respectively.^38^

Thermogravimetric analysis (TGA) was performed in air to confirm the carbon content of the I–S@MCNT composite. As shown in Figure 2b, the TGA curves of I–S and I–S@MCNTs showed weight losses of about 20.9% and 29.9%, respectively, over the temperature range of 100–700 °C. These weight losses originate from two reactions: (1) the conversion of indium and antimony sulfides into their oxides, which occurred in both samples;^39^ and (2) gas produced by combustion of MCNTs, which only occurred for the I–S@MCNTs. The MCNT content determined from the TGA results is ≈9.0%, which is a reasonable value to obtain a balance between energy density and conductivity.

X‐ray photoelectron spectroscopy (XPS) was used to verify the presence of In, Sb, S, C, and O in the I–S@MCNTs, as shown in Figure 2c. In the Sb 3d spectrum (Figure 2d), the characteristic peaks at 529.8 and 539.2 eV are assigned to Sb 3d_5/2_ and Sb 3d_3/2_, respectively, indicating the presence of Sb^3+^. Furthermore, the O 1s peak appears in the Sb 3d spectrum, which likely originates from oxygen‐containing functional groups on the MCNTs. These functional groups can effectively improve the sodium storage capacity of the material through surface redox reactions.^22^ The characteristic peaks at 445.1 and 452.6 eV in Figure 2e correspond to the In 3d_5/2_ and In 3d_3/2_ signals of In^3+^, respectively.^37^ The S 2p spectrum (Figure 2f) shows peaks near 161.5 and 162.9 eV, which we ascribe to S 2p_3/2_ and S 2p_1/2_, respectively. This spectrum can be further fitted into four peaks originating from S atoms in various functional groups, including single doublets from S—Sb and In—Sb bonds. Meanwhile, the chemical interaction between MCNTs and I–S was confirmed by the existence of a signal from S=C=S bonds.^40^ As shown in Figure S6 of the Supporting Information, a signal corresponding to double bonds in the C 1s spectrum also indicated the combination of I–S and MCNTs. All of the measured physiochemical properties of the I–S and I–S@MCNT samples suggest that they contain a heterostructure of In_2_S_3_ and Sb_2_S_3_ with a formicary structure.

**Figure 3:**
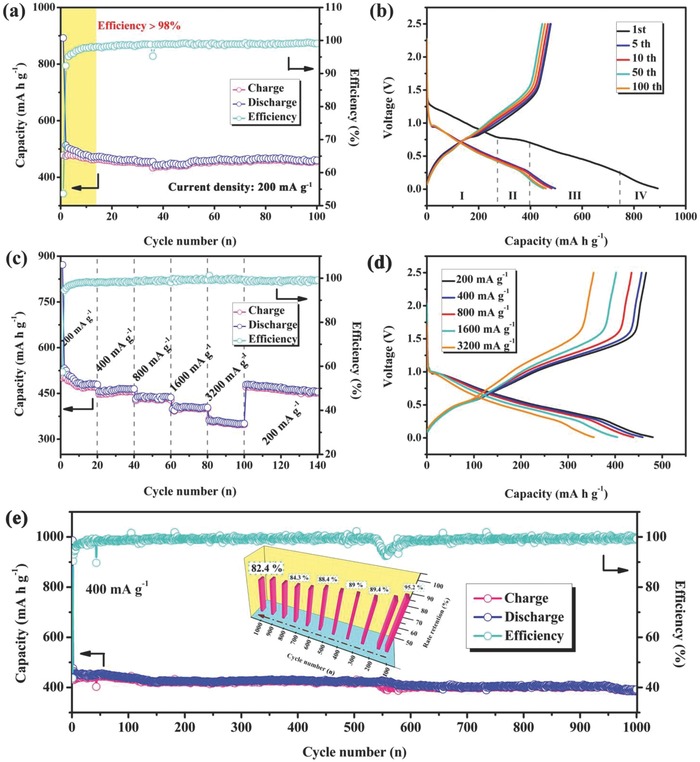
a) Cycle performances of I–S and I–S@MCNTs electrodes. b) Charge/discharge curves of I–S and I–S@MCNTs in the voltage range of 0.01–2.5 V at current density of 200 mA g^−1^. c) Rate performances of I–S and I–S@MCNTs electrodes. d) Charge/discharge curves of I–S@MCNTs at different current densities. e) Long cycle performance of I–S@MCNTs electrode tested at current density of 400 mA g^−1^, inset is the capacity retention histogram.

The electrochemical performance of an I–S@MCNT electrode was evaluated by measuring galvanostatic charge/discharge curves. As shown in **Figure** **3**a, the electrode displayed a reversible charge capacity of 470 mA h g^−1^ at a current density of 200 mA g^−1^, with a high coulombic efficiency of above 98% after 13 cycles. The capacity contribution of the MCNTs to the total capacity of the I–S@MCNT electrode was very limited (Figure S7a, Supporting Information) because of their low electrochemical activity (Figure S7b, Supporting Information) and small content in the electrode. A reference bare Sb_2_S_3_ electrode exhibited rapid capacity fading, as shown in Figures S8a and S9a of the Supporting Information. It is speculated that a synergistic effect of Sb_2_S_3_ and In_2_S_3_ enhances the cycling stability of the I–S@MCNT electrode. The lower specific capacity of the I–S@MCNT electrode compared with that of the Sb_2_S_3_ electrode can be ascribed to the formation of inert components during the cycling process, which consumed a certain amount of active components.

The charge/discharge profiles of the I–S@MCNT electrode during the first cycle (Figure 3b) are similar to the cyclic voltammetry (CV) curves and can be divided into four parts, which demonstrate a sequential sodium storage process involving intercalation, conversion, and alloying. Note that both Sb^3+^ and In^3+^ showed electrochemical activity in the sodium storage reaction, meaning that two pairs of Na^+^ can be captured during the insertion and extraction process. However, the sodiation and the desodiation of In_2_S_3_ (Figure S10, Supporting Information) occur at potentials close to those of Sb_2_S_3_. Subsequently, the In‐containing components gradually converted into inert components, resulting in the existence of only one group of redox peaks in CV curves. Furthermore, the voltage plateaus in the charge/discharge profiles after 100 cycles were perfectly maintained without increasing polarization voltage. However, the increase in polarization voltage between the charge and discharge curves of the In_2_S_3_ electrode (Figure S9b, Supporting Information) may be the immediate reason for its poor cycling performance.

The I–S@MCNT electrode exhibited a rate capability of up to 3.2 A g^−1^, as shown in Figure 3c. The reversible capacities stabilized near 467, 452, 431, and 402 mA h g^−1^ at current densities of 200, 400, 800, and 1600 mA g^−1^, respectively. A specific capacity of 355 mA g^−1^ was obtained at an ultrahigh current density of 3.2 A g^−1^. When the current density was returned to 200 mA g^−1^, the capacity fully recovered to 454 mA h g^−1^ after 40 cycles. Figures S8b and S9c of the Supporting Information compare the capacities of I–S@MCNT, Sb_2_S_3_, and In_2_S_3_ electrodes at different current densities, revealing the remarkably enhanced rate performance of the I–S@MCNT electrode compared with those of the other electrodes.

As the current density was increased from 200 to 3200 mA g^−1^, the charge and discharge profiles of the I–S@MCNT electrode (Figure 3d) remained similar, suggesting that the composite wrapped with MCNTs had fast migration rates of Na^+^ and electrons (Figure S11, Supporting Information). However, the starting voltages of the discharge and charge profiles of the In_2_S_3_ electrode (Figure S9d, Supporting Information) were quite different at 1.58 and 0.1 V, respectively, deviating considerably from the set values. After 1000 cycles (Figure 3e), the In_2_S_3_ electrode still showed a considerable capacity of ≈400 mA h g^−1^ at a current density of 400 mA g^−1^, retaining 84.2% of its initial charge capacity. The cycling stability and rate performance of the I–S@MCNT electrode and other Sb_2_S_3_‐based electrodes are compared in Table S2 (Supporting Information), illustrating the considerable improvement of electrochemical properties achieved for the I–S@MCNT electrode. The In_2_S_3_–Sb_2_S_3_ composite plays a critical role in enhancing the long‐term cyclability and rate performance of the I–S@MCNT electrode.

The I–S@MCNT electrode also exhibited outstanding performance as an anode for LIBs (Figure S12a, Supporting Information), delivering a reversible charge capacity of 613 mA h g^−1^ after 100 cycles. The charge/discharge profiles of the I–S@MCNT electrode in an LIB (Figure S12b, Supporting Information) indicate a similar lithium storage process to that of sodium storage, consisting of intercalation, conversion, and alloy reactions. After long‐term cycling, we measured galvanostatic intermittent titration technique (GITT) curves for the I–S@MCNT electrode (Figure S13a, Supporting Information), which showed that its charge/discharge profiles were close to thermodynamic equilibrium.^23^, ^41^ The high average sodium storage potential of the I–S@MCNT electrode (above 0.5 V) prevents the hazard of sodium dendrites, making it a safe anode for SIBs. The GITT results revealed that the I–S@MCNT electrode exhibited considerable chemical diffusion coefficients even after 600 cycles (Figure S13b, Supporting Information), which lead to high ionic conductivity.

**Figure 4:**
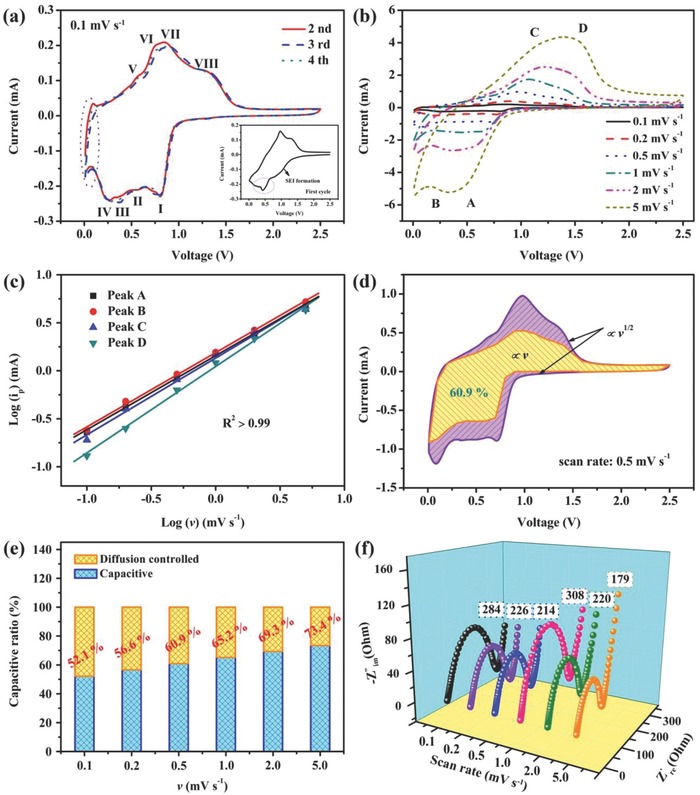
Kinetic analysis of sodium storage in I–S@MCNTs electrode: a) CV curves of I–S@MCNTs electrode at 2nd, 3rd, and 4th cycles, the first cycle was described in the inset. b) CV curves of I–S@MCNTs electrode at various scan rates from 0.1 to 5 mV s^−1^. c) Linear plot of the relationship between log(Ip) and log(v) for both the anodic and cathodic scans of the I–S@MCNTs electrode. d) Capacitive contribution (yellow area) and diffusion contribution (purple area) for working current obtained at a scan rate of 0.5 mV s^−1^. e) Normalized contribution ratio of capacitive capacities at different scan rates. f) EIS of I–S@MCNTs electrode after corresponding scan rates.

A series of electrochemical experiments was carried out to investigate the Na^+^ storage kinetics of the I–S@MCNT electrode. As shown in the inset of **Figure** **4**a, the CV curve of the first cycle displayed a large reduction peak in the potential region of 0.65–1.35 V, indicating the formation of a solid electrolyte interface film on the electrode surface. At the same time as this irreversible passivation reaction, Na^+^ was inserted into the host structure of I–S. The conversion reaction between Na_2_S, Sb, In, and I–S then occurred near 0.5 V. The obvious reduction peak at 0.2–0.4 V may originate from conversion and alloying during the formation of Na*_x_*Sb.^24^, ^37^ Scanning in the reverse direction gave four anodic peaks related to reversible sodium extraction. However, the redox peaks in later cycles were quite different from those of the first cycle. We attribute this difference to changes in the composition of the electrode during cycling. Among these peaks, those labeled as I and V; II and VI; and III, IV, VII, and VIII are related to Na insertion, conversion, and alloying reactions, respectively. The sharp redox peaks at lower potential originate from sodium storage in the MCNTs.^42^ The good reproducibility of the CV curves during continuous charging and discharging shows that the electrode has both highly reversible structure and reaction activity.

To investigate the sodium storage mechanism in the I–S@MCNT electrode, we measured CV curves at different scan rates, which revealed two pairs of redox peaks (Figure 4b). Cathodic peak A corresponds to the intercalation of Na^+^ into the Sb_2_S_3_ layered structure. Accordingly, anodic peak D corresponds to the deintercalation of Na^+^ from Sb_2_S_3_. Peaks B and C originate from conversion and alloying reactions with similar potentials. These well‐defined redox peaks reveal that the sodiation and the desodiation reactions on the electrode are the same at different scan rates. As shown in previous work,^43^, ^44^ the capacitive contributions on the electrode surface to the total sodium storage capacity can be qualitatively confirmed by assessing the relationship between the scan rate (*v*) and recorded current (*i*) from CV curves measured at different sweep rates

(1) logi=blogv+loga

Here, the slope *b* determined by the linear relationship between log *v* and log *i* describes the ionic storage process of the electrode. For a diffusion‐controlled process, *b* approaches 0.5, whereas it approaches 1 for a capacitance‐dominated process. Therefore, the high *b* values of the I–S@MCNT electrode (0.771, 0.774, 0.8, and 0.9 for peak A, B, C, and D, respectively) demonstrate its favorable capacitive kinetics (Figure 4c). The pseudocapacitive characteristics of the electrode are reflected in the slow change of the normalized capacity as the scan rate increases from 0.1 to 1 mV s^−1^ (Figure S14, Supporting Information).

The current response at a specific potential has contributions from diffusion insertion and surface capacitive effects

(2) $$i=k_{1}v+k_{2}v^{1/2}$$

Here, *k*_1_ *v* and *k*_2_ *v* ^1/2^ represent the contributions to the current from the capacitance‐dominated process and intercalation‐controlled process, respectively. The values of *k*_1_ and *k*_2_ can be uniquely determined at a fixed potential. The intercalation contribution is dominant near the voltage range of the redox peak, where the redox reactions of Sb^3+^/Sb^0^ and In^3+^/In^0^ may promote sodium diffusion.^45^ Consequently, the corresponding current contribution for the pseudocapacitance‐dominated process can be quantitatively calculated from the potential. Figure 4d shows the relationship between total stored charge and capacitive stored charge, which indicates that 60.9% of the total charge is capacitive at a scan rate of 0.5 mV s^−1^. In addition, the capacitive contribution ratios at other scan rates were also calculated, as shown in Figure 4e. The capacitive contribution strongly depends on scan rate, increasing from 52.1% of the total capacity at 0.1 mV s^−1^ to a maximum of 73.4% at 5 mV s^−1^. Thus, we infer that the outstanding rate performance of the I–S@MCNT electrode originates from its pseudocapacitance effect that arises from its large surface area and abundant pores, which provide many active sites and fast sodium storage kinetics.

The enhanced reaction kinetics of the I–S@MCNT electrode caused by the honeycomb structure and high conductivity of the MCNTs was also verified by electrochemical impedance spectroscopy (EIS), as depicted in Figure 4f. The EIS curve consisted of a flat semicircle in the high‐frequency region and a curved line in the low‐frequency region. According to the equivalent circuit proposed in another report,^46^ the surface resistance and charge‐transfer resistance of the I–S@MCNT electrode at different scan rates were confirmed to be low (in the range of 200–300 Ω), indicating superior electron transport and reaction features compared with those of the In_2_S_3_ electrode (Figure S15, Supporting Information).

**Figure 5:**
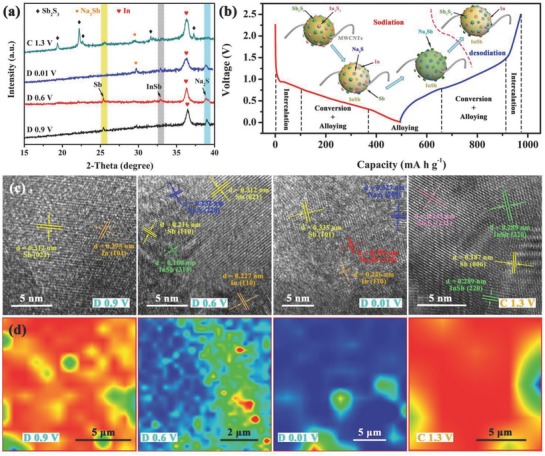
Ex situ measurements for I–S electrode during the charging–discharging process: a) Ex situ XRD patterns captured at different potentials. b) Charging–discharging profiles of the third cycle at 100 mA g^−1^, insets are structure evolution illustration of I–S. c) Ex situ TEM and corresponding HRTEM images, insets in HRTEM images are relative FFT patterns. d) Ex situ Raman mapping of I–S electrode.

We investigated the sodium storage mechanism of the I–S@MCNT electrode in detail by conducting various ex situ measurements. When the voltage was decreased to 0.9 V (named “D0.9 V”), the conversion reaction generated metallic Sb, metallic In, and Na_2_S, as shown by the peaks at 25.6°, 36.3°, and 38.9°, respectively, in the ex situ XRD patterns (**Figure** **5**a).^47^ Note that a characteristic peak at 33° appeared when the cell was discharged to 0.6 V (named “D0.6 V”), indicating the formation of an InSb phase with the *I41/amd* space group. This phase can effectively alleviate the influence of volume expansion during cycling because of its electrochemical inertia in Na^+^ storage. When the cell has been fully discharged (named “D0.01 V”), its faint lattice fringes indicate that the discharge products have low crystallinity. The peak at 29.7° suggests that an alloying reaction occurs between sodium and antimony to form Na_3_Sb.^15^ During reverse charging (the XRD pattern was measured at ≈1.3 V), Sb_2_S_3_ was gradually regenerated with high reversibility. In particular, the intense peak at 36.3° did not disappear during Na^+^ extraction, showing that the partially metallic indium transformed into irreversible components. These components played a crucial role as a buffer layer, preventing the material from being pulverized by the huge volume expansion during sodiation/desodiation. This phenomenon can explain the large difference between the first and second CV curves, which originates from the different chemical compositions of the electrode in the first and second cycles.

The active antimony and inert indium in the I–S@MCNT electrode were also verified by XPS. The peak at 768.2 eV (Figure S16a, Supporting Information) gradually disappeared during discharging and then appeared again in the reverse process, suggesting a reversible valence change of the Sb^3+^/Sb^0^ couple. The peaks at binding energies of 452.6 and 445 eV (Figure S16b, Supporting Information) are related to the 3d_3/2_ and 3d_5/2_ levels of In^3+^, respectively. The binding energies of these peaks decreased by 0.5 eV during discharging, indicating the reduction of In^3+^. The In^0^ signal was always observed in successive sodiation and desodiation reactions, indicating that some indium formed irreversibly in the electrode. Considered together, the results indicate that the storage and extraction of Na^+^ in the I–S@MCNT electrode involves a three‐step process of intercalation, conversion, and alloying (Figure 5b). The inset of Figure 5b shows the various electrode compositions at different voltage ranges, corresponding to the presence of reversible and irreversible products.

To directly observe these products, especially for the weakly crystalline compounds, HRTEM images (Figure 5c) were collected at the four potentials mentioned above. In the “D0.9 V” sample, the *d* spacings of 0.312 and 0.275 nm are consistent with the (021) and (101) crystal planes of metallic antimony and indium, respectively. The InSb phase appeared between the two metallic antimony phases, which relieved the volume expansion and reciprocal extrusion of sodiated antimony. The HRTEM image of the “D0.01 V” sample reveals the presence of Na_3_Sb formed by full alloying of Sb, as demonstrated by the (311) fringes for Na_3_Sb. When the I–S@MCNT electrode was charged to 1.3 V, clear (231) fringes of Sb_2_S_3_ with high crystallinity reappeared in the HRTEM image because of the highly reversible conversion reaction. The InSb phase retained in the gaps between Sb_2_S_3_ provides an effective buffer for subsequent conversion and alloying reactions.

Figure 5d shows the Raman mappings that indicate the intensity of Sb—Sb bonds in electrodes at different states, revealing their chemical compositions and interface states.^48^ The highly reversible Na^+^ storage process can be verified by the reappearance of metallic Sb. Based on the low crystallinity observed in the XRD patterns, the existence of intermetallic InSb is demonstrated in the Raman spectra (Figure S17, Supporting Information), which show characteristic peaks of the In—Sb bond at 149.1 and 465.8 cm^−1^. When the electrode was fully discharged, the Sb—Sb bonds gradually disappear as Na_3_Sb forms. The Raman spectra were reproducible for the same electrode in different regions, indicating a uniform passivation layer formed on the electrode surface.

**Figure 6:**
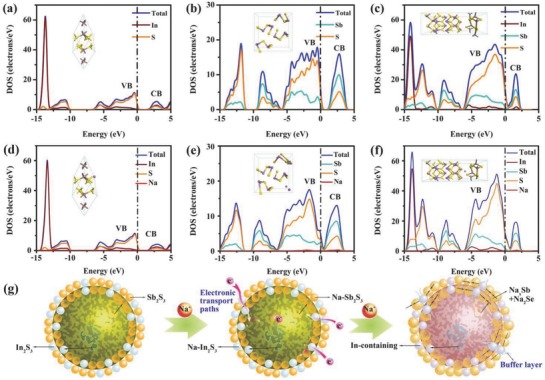
The partial density states (PDOS, marked by the line of brown for In, green for Sb, orange for S, red for Na) and total density of states (TDOS, marked by the line of blue) of a) In_2_S_3_, b) Sb_2_S_3_, c) heterostructure of I–S and d) Na‐inserted In_2_S_3_, e) Na‐inserted Sb_2_S_3_, f) Na‐inserted I–S. Insets are corresponding crystal structures of these compounds. g) Schematic illustration of the buffer action and conductive function of In‐containing layer.

To better understand the enhanced electrochemical performance in the heterostructure of In_2_S_3_ and Sb_2_S_3_, we used density functional theory (DFT) to investigate the energy band structures and electronic cloud distributions of In_2_S_3_, Sb_2_S_3_, I–S, and their Na‐inserted structures. The insets of **Figure** **6** show optimized model diagrams of these materials. The band gap refers to the distance between the discrete valence band (VB) and conduction band (CB). From −15 to 5 eV, the density of states (DOS) patterns and partial density of states (PDOS) patterns of In, Sb, and S in I–S (Figure 6c) exhibit a much narrower band gap (0.116 eV) than those of Sb_2_S_3_ (1.458 eV, Figure 6a) and In_2_S_3_ (0.663 eV, Figure 6b), revealing that the heterostructure has improved electron conductivity. This phenomenon can be attributed to the delocalized In 3d and Sb 3d orbitals, which widen the VB and CB and thus narrow the band gap.^49^ Moreover, the Femi level shifted to the CB, suggesting that the primary carriers in this structure were electrons.

The band gaps of Sb_2_S_3_ (Figure 6e) and I–S (Figure 6f) gradually narrowed to 0.913 and 0.103 eV, respectively, when Na^+^ was inserted into the host structures. We attribute this behavior to the increased overlap of the electronic orbitals of Sb, S, and Na, indicating the high activity of Sb_2_S_3_ and I–S toward Na^+^. By contrast, the band gap of In_2_S_3_ widened following Na^+^ insertion (Figure 6d), revealing lower activity toward Na^+^ and a stable sodium‐storage structure. Moreover, the energy barrier of I–S (3.8323 eV) was slightly lower than those of In_2_S_3_ (3.9352 eV) and Sb_2_S_3_ (3.9926 eV), as confirmed by the formula *E*_barrier_ = *E*_sodiated electrode_ – (*E*_electrode_ + *E*_Na+_). Table S3 (Supporting Information) presents detailed energy data for these compounds.

The In‐containing components act as electronic transport paths and buffer layers in the I–S@MCNT electrode during insertion/extraction of Na^+^, as shown in Figure 6g. In the intercalation reaction, the volume of the Sb_2_S_3_ phase expands more than that of the In_2_S_3_ phase, meaning that the stable structure of Na‐inserted In_2_S_3_ appears to act as a support skeleton between Sb_2_S_3_ phases. In the alloying reaction, the remarkable volume change of metallic Sb and Na_3_Sb can be relieved by the presence of the metallic In phase, which is an inert constituent of the electrode. Therefore, the In‐containing components improved the cycling stability of the I–S@MCNT electrode.

## 3 Conclusion

Using various methods, we investigated the electrochemical performance and novel sodium storage process of an I–S@MCNT anode for SIBs, which represents a new electrode design. The I–S@MCNT electrode showed a reversible specific capacity of 400 mA h g^−1^ at a current density of 400 mA g^−1^ after 1000 cycles. Even at a high rate of 3200 mA g^−1^, the electrode maintained a current density of 355 mA h g^−1^. We ascribe the favorable kinetic properties of the I–S@MCNT electrode to its optimized structure and the inclusion of MCNTs. Therefore, modifying metal sulfides in this manner is promising for the following three reasons: (1) it creates interfaces and a porous morphology, (2) it introduces an electrochemically inert component (such as In_2_S_3_) to support the structural stability of the electrode during redox reactions, and (3) it lowers the Na^+^ migration barrier by improving the electronic structure of the electrode. In addition, the poor conductivity of the sulfide nanostructure can be enhanced by coating with a carbon material.

## 4 Experimental Section

*Synthesis*: The I–S microspheres were synthesized by the following solvothermal method. Thiocarbamide (0.01 mol) was dissolved in ethanediol (80 mL) under violent agitation to form solution A. Meanwhile, SbCl_3_ (0.0016 mol) and an equimolar amount of InCl_3_ were dissolved in stirred ethanediol (80 mL) to form solution B. Solution A was rapidly added to solution B, and then the resulting mixture was stirred for 4 h. The solution was transferred to a 200 mL stainless steel Teflon‐lined autoclave and then reacted at 200 °C for 32 h. After the autoclave had cooled naturally to room temperature, the reddish‐brown precipitate was collected by high‐speed centrifugation, washed repeatedly with absolute ethanol and deionized water, and then dried under vacuum at 80 °C overnight.

To improve the electronic conductivity of I–S, we formed an in situ composite of the active materials with MCNTs as follows. MCNTs (50 mg) were dispersed in solution B by ultrasonication for 1 h and then stirred for 2 h to produce MCNTs with attached metal ions. Subsequent mixing of solutions and processing were conducted as described above, providing I–S@MCNT.

*Material Characterization*: Wide‐angle XRD patterns were recorded with a powder X‐ray diffractometer (Hitachi Rigaku‐D/Max‐2550 PC, Japan) over the 2θ range of 10°–70°. The specific surface area of I–S was determined by N_2_ adsorption–desorption measurements using a micropore analyzer (ASAP‐2460). Sample morphology and microstructure were observed by SEM (Hitachi, S‐4800, Japan). TEM and HRTEM images were obtained using a JEOL JEM‐2100F electron microscope. The element distribution was determined by EDS with an Oxford attachment. Exact elemental contents were confirmed by ICP‐AES (Vista‐MPX, America). XPS (PHI Quantera‐II, Japan) was conducted using monochromatic Al Kα radiation. Raman spectra and images were obtained with a Thermo Fisher DXR2xi spectrometer. The MCNT content was measured by TGA, which was performed with a simultaneous thermal analyzer (Netzsch STA449F3, Germany) in air with a temperature ramp of 10 °C min^−1^. The cycled electrodes were repeatedly washed with volatile dimethyl carbonate to remove the passivation layer. The electrodes and mixed powders were studied with XRD, Raman spectroscopy, TEM, and XPS.

*Electrochemical Measurements*: All electrochemical tests were performed using two‐electrode systems assembled as 2032‐type coin cells. A powder of as‐prepared materials (70 wt%) and active carbon (Super P; 20 wt%) was bonded with carboxymethylcellulose sodium binder (10 wt%) and then coated on copper foil. The resulting electrode films were dried in a vacuum oven at 60 °C for 24 h, and then cut into 1 × 1 cm^2^ squares with a mass loading of ≈1.5 mg cm^−2^. Metallic Na was used as the counter electrode and a glass fiber filter (Whatman GF/C) was used as the separator. The electrolyte was composed of 1 m NaClO_4_ and equal volumes of ethylene carbonate and diethyl carbonate containing 5% FEC. Galvanostatic charge/discharge data were measured with a battery tester (Land, Wuhan Kingnuo Electronics Co. Ltd, Wuhan, China) over a voltage range of 0.01–2.5 V versus Na^+^/Na. CV and EIS measurements were conducted on an electrochemical analyzer (Shanghai Chenhua Instrument Co. Ltd, Shanghai, China) in the frequency range of 10 kHz to 0.1 Hz.

*Computation Methods*: Theoretical investigations were performed using DFT as implemented in the plane wave code Cambridge sequential total energy package (CASTEP).^50^, ^51^ The generalized gradient approximation was used to treat the exchange‐correlation functions. The kinetic energy cutoff for the plane wave expansion was set to 450 eV for later calculations in reciprocal space,^52^ which is high enough for 2D layered materials. The optimized models of monocrystalline Sb_2_S_3_ with an orthorhombic structure and In_2_S_3_ with a rhombohedral structure were used to calculate properties after transforming them into 2 × 2 × 2 supercells. The I–S heterostructure was constructed by stitching together the (100) crystal plane of Sb_2_S_3_ and (121) crystal plane of In_2_S_3_ without a redundant vacuum layer, producing a heterostructure with well‐defined periodicity. A k‐point mesh density of 5 × 5 × 5 was used for integration over the Brillouin zone. To obtain accurate results, we used a maximum self‐consistent field convergence threshold of <5 × 10^−6^ eV atom^−1^. The other convergence tolerances for the maximum force, maximum stress, and maximum displacement were set to 0.01 eV Å^−1^, 0.02 GPa, and 5 × 10^−4^ Å, respectively. In particular, the calculations of the total DOS and PDOS used a bond‐energy tolerance of 1 × 10^−5^ eV.

## Conflict of Interest

The authors declare no conflict of interest.

## Supporting information

Supplementary
