# Supplementary material for: Conductivity and Pseudocapacitance Optimization of Bimetallic Antimony–Indium Sulfide Anodes for Sodium‐Ion Batteries with Favorable Kinetics
Source: Adv Sci (Weinh). 2018 Jul 26;5(10):1800613. doi: 10.1002/advs.201800613 (PMC6193174; doi:10.1002/advs.201800613)
Supplement: Supplementary file 1 — Supplementary [file ADVS-5-1800613-s001.pdf]

Copyright WILEY-VCH Verlag GmbH & Co. KGaA, 69469 Weinheim, Germany, 2018.

Supporting Information

for *Adv. Sci.*, DOI: 10.1002/advs.201800613

Conductivity and Pseudocapacitance Optimization of Bimetallic Antimony–Indium Sulfide Anodes for Sodium-Ion Batteries with Favorable Kinetics

*Yongxin Huang, Ziheng Wang, Ying Jiang, Shuaijie Li, Min Wang, Yusheng Ye, Feng Wu, Man Xie, Li Li, and Renjie Chen**

Supporting Information

Conductivity and Pseudocapacitance Optimization of Bimetallic Antimony–Indium Sulfide Anodes for Sodium-ion Batteries with Favorable Kinetics

*Yongxin Huang, Ziheng Wang, Ying Jiang, Shuaijie Li, Min Wang, Yusheng Ye, Feng Wu, Man Xie, Li Li, and Renjie Chen**

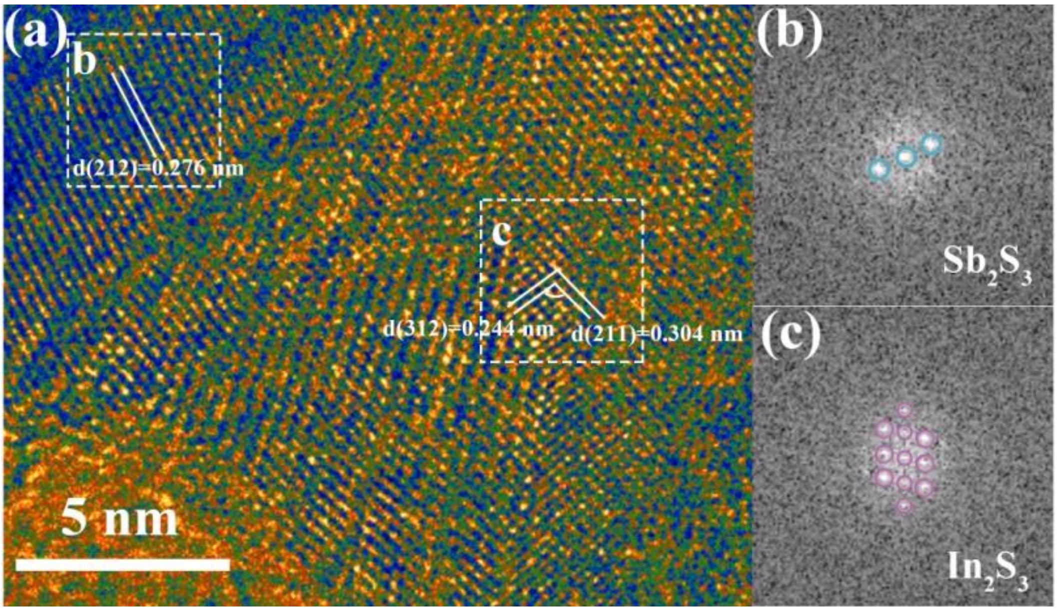

Figure. S1 (a) HRTEM image of In_2S_3-Sb_2S_3 microspheres; the corresponding fast Fourier transform (FFT) patterns of marked areas in the former, which belong to orthorhombic Sb_2S_3 phase (b) and rhombohedral In_2S_3 phase (c), respectively.

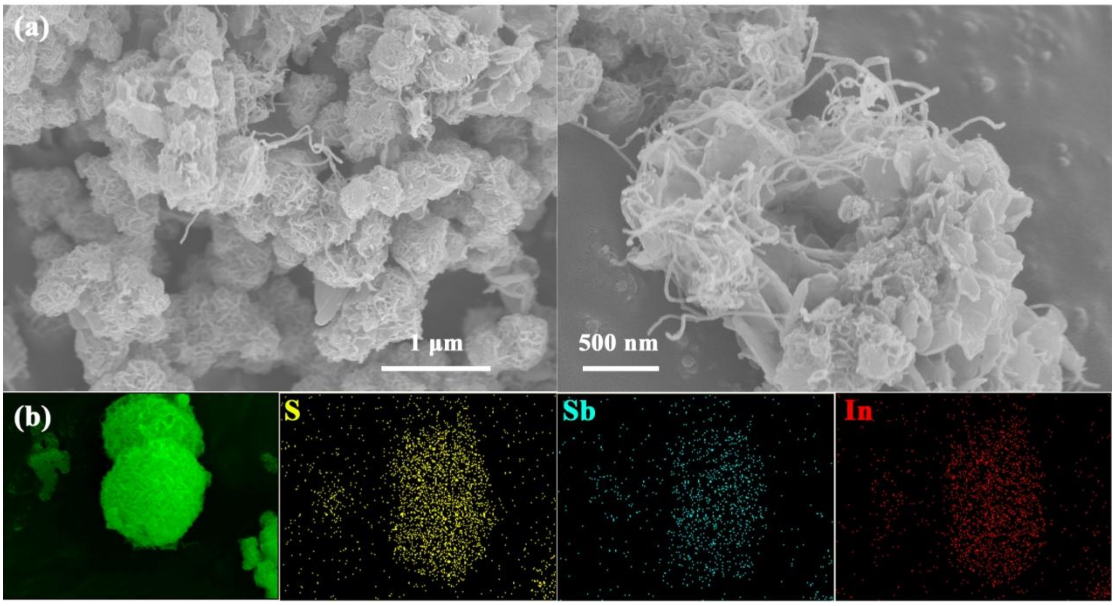

Figure. S2 (a) SEM images of I-S@MWCNTs sample with different magnifications; (b) EDX mapping images of I-S@MWCNTs sample with corresponding SEM image.

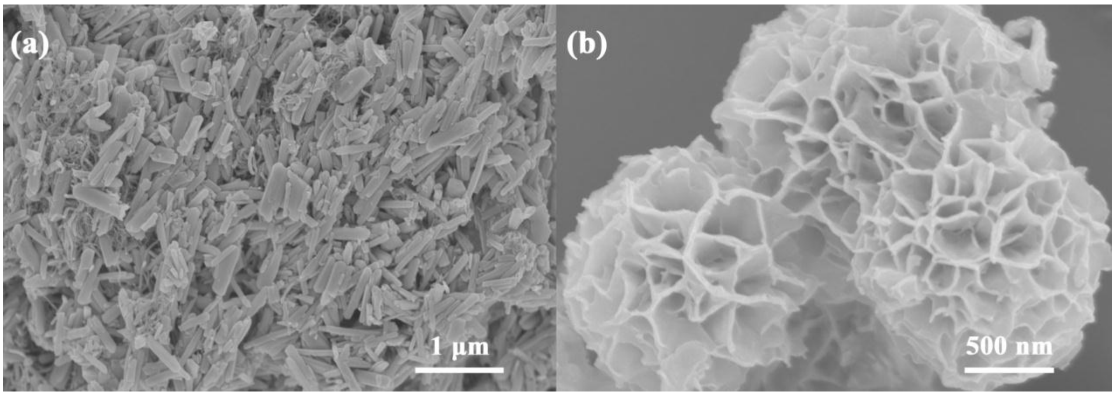

Figure. S3 SEM images of rod-like Sb_2S_3 (a) and flower-like In_2S_3 (b) synthetized via the similar hydrothermal routes.

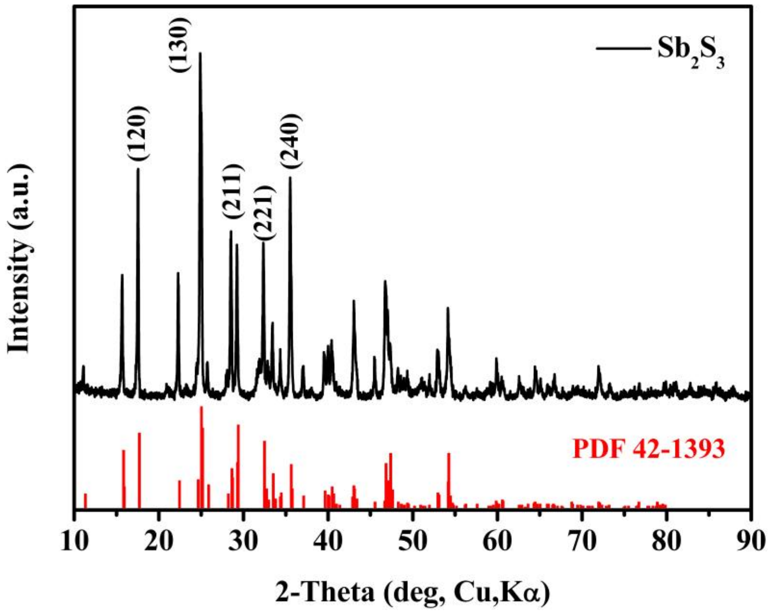

Figure. S4 XRD pattern of bare Sb_2S_3 sample synthetized by a similar approach.

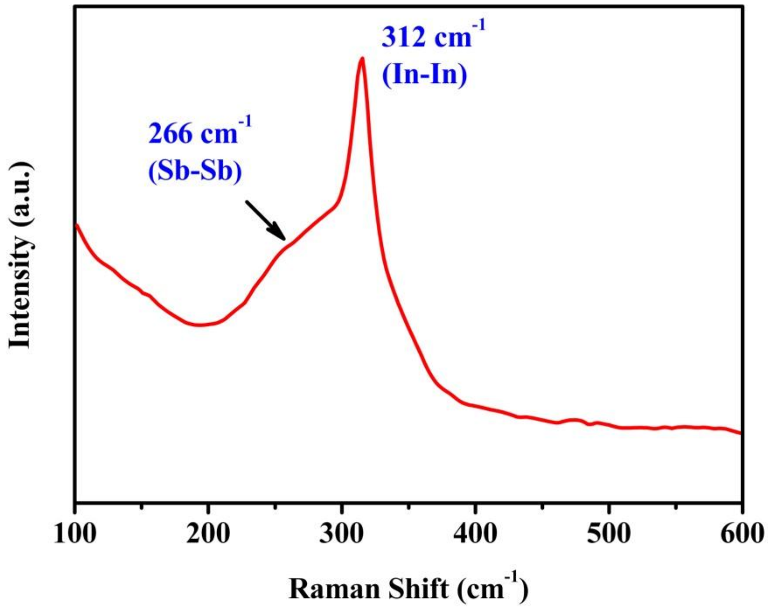

Figure. S5 Raman spectrum of the original I-S@MWCNTs sample.

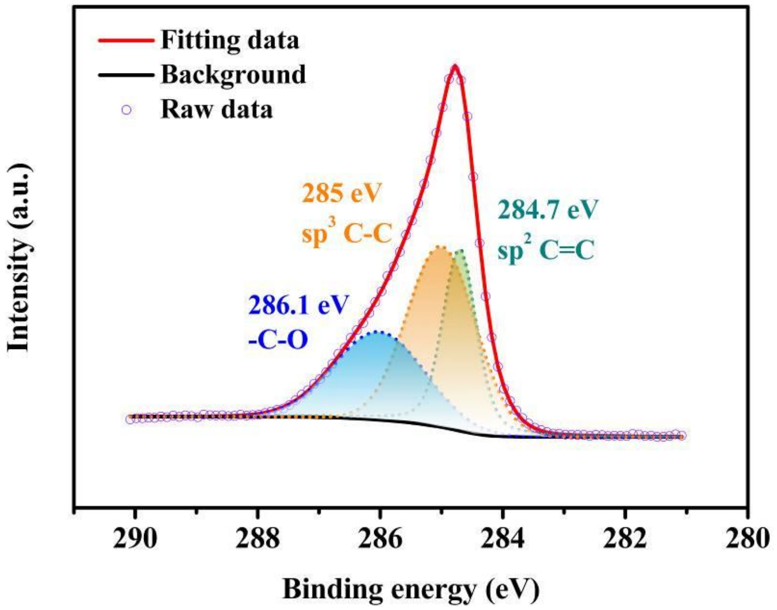

Figure. S6 C 1s high-resolution XPS of the I-S@MWCNTs composite.

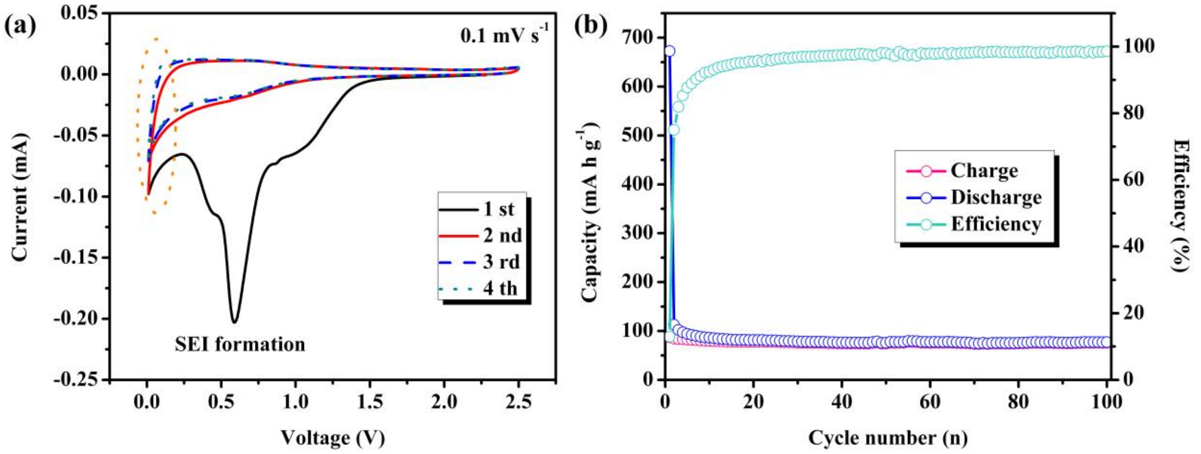

Figure. S7 Electrochemical behaviors of MWCNTs electrodes prepared by mixing MWCNTs (70 wt. %), conductive agent (20 wt. %) and binder (10 wt. %): (a) CV curves at scan rate of 0.1 mV S^{-1}; (b) Cycling performance at 200 mA g^{-1} between voltage range of 0.01 – 2.5 V.

As shown in Figure. S6a, there is a sharp peak at 0.01 V in CV curves, indicating the low sodium storage activity of MWCNTs electrode. This phenomenon also illustrated that the electrochemical behaviors of I-S sample could be observed without the interference from MWCNTs. The MWCNTs electrode exhibited a capacity of 80 mA h g^{-1} (Figure. S6b), when it was used as activity materials. If it is used as composite accounting for 9 wt. % of the active materials, it only delivered a low capacity of ~7 mA h g^{-1}, which is negligible for total capacity.

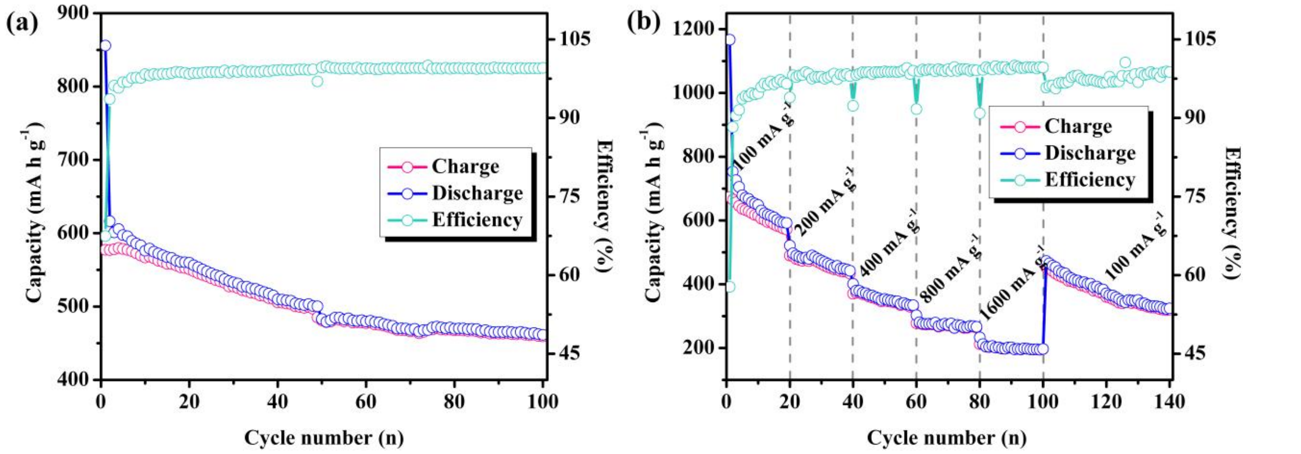

Figure. S8 Na-storage performances of Sb_2S_3 electrode synthetized by similar condition: (a) Cycling performance at 200 mA g^{-1} between voltage range of 0.01 – 2.5 V; (b) Rate performances at various current density.

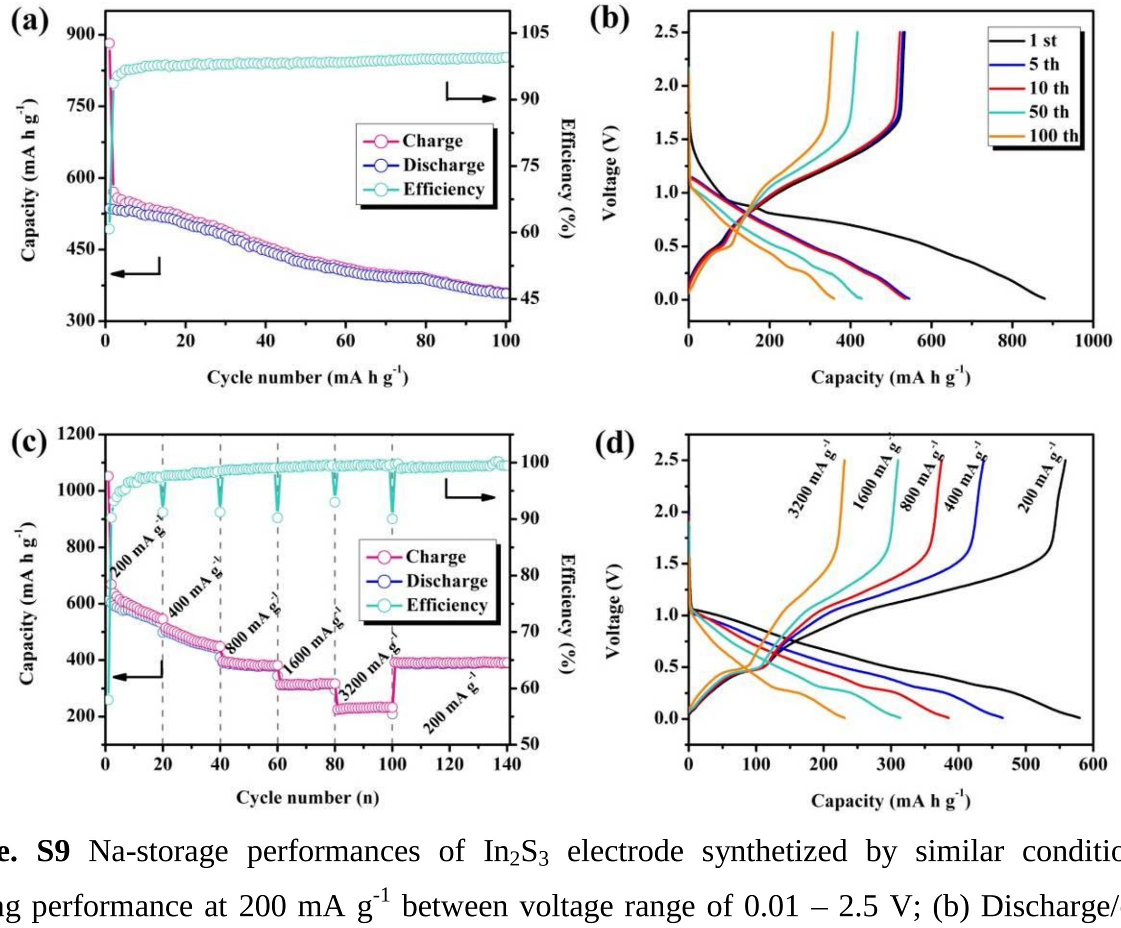

Figure. S9 Na-storage performances of In_2S_3 electrode synthetized by similar condition: (a) Cycling performance at 200 mA g^{-1} between voltage range of 0.01 – 2.5 V; (b) Discharge/charge curves at different cycles at 200 mA g^{-1}; (c) Rate performances at various current density; (d) Initial discharge/charge curves at various current densities.

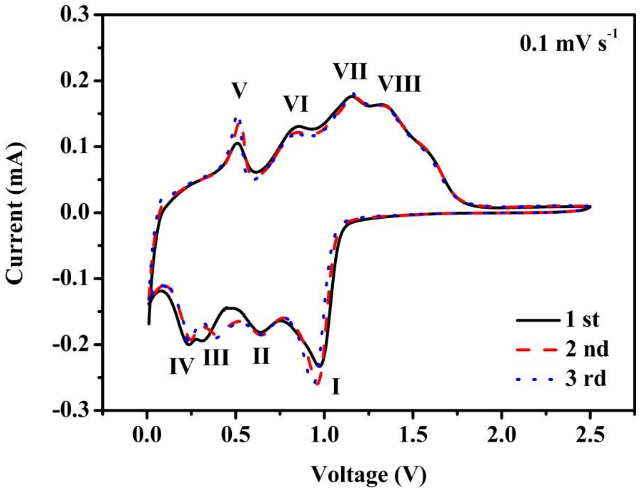

Figure. S10 CV curves of In_2S_3 electrode at scan rate of 0.1 mV s^{-1}.

The electrochemical activity and reaction mechanism of In_2S_3 electrode for sodium storage was revealed by the CV curves tested at 0.1 mV s^{-1}. Among them, the peak I and V correspond to the insertion and extraction process of Na^+ ions in In_2S_3. The peak II can be described as the conversion reaction from In_2S_3 to In and Na_2S. Accordingly， the peak VI corresponds to the inverse process of conversion reaction. The peak III and IV represent the stepwise conversion and alloy reaction between metallic In, Na_2S and In-Na alloy. While the symmetrical peak VII and VIII indicated the complete recovery of In_2S_3 electrode. Especially, the positions of these peaks are close to the characteristic peaks of sodium storage in Sb_2S_3.

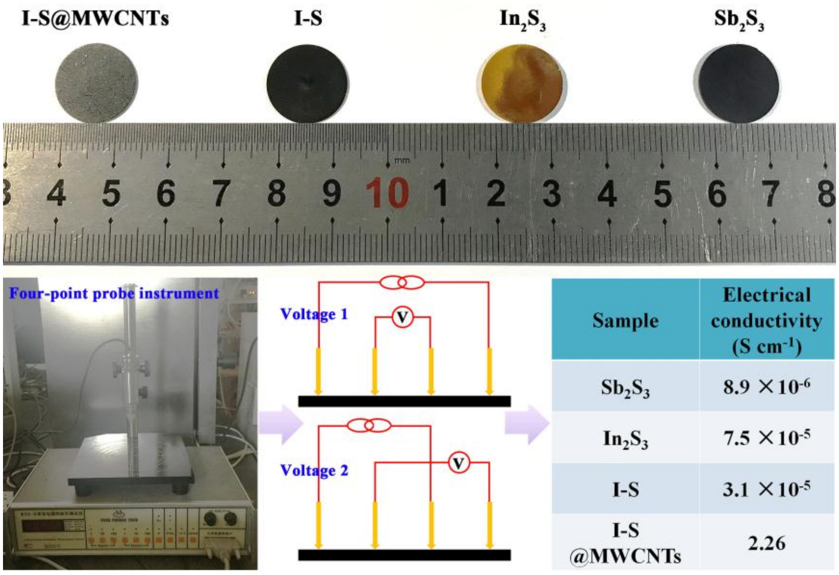

Sample	Electrical conductivity (S cm^{-1})
Sb_2S_3	8.9×10^{-6}
In_2S_3	7.5×10^{-5}
I-S	3.1×10^{-5}
I-S @MWCNTs	2.26

Figure. S11 Electrical conductivities of Sb_2S_3, In_2S_3, I-S and I-S@MWCNTs samples, which are pressed into pieces and then measured on a four-point probe instrument.

The electrical conductivities of Sb_2S_3, In_2S_3, I-S and I-S@MWCNTs were measured via a four-point probe method. Firstly, these samples were pressed into pieces with a diameter of 1.5 cm, which presented different colors. Subsequently, these pieces were closely contacted to the instrument, and the corresponding currents were adjusted to the appropriate values. Finally, the electrical conductivities can be calculated according to the voltages. The I-S@MWCNTs exhibits an outstanding electrical conductivity of 2.26 S cm^{-1}, which is approximately to the pure MWCNTs. On the other hand, the In-doping improved the electrical conductivity of bare Sb_2S_3 simultaneously.

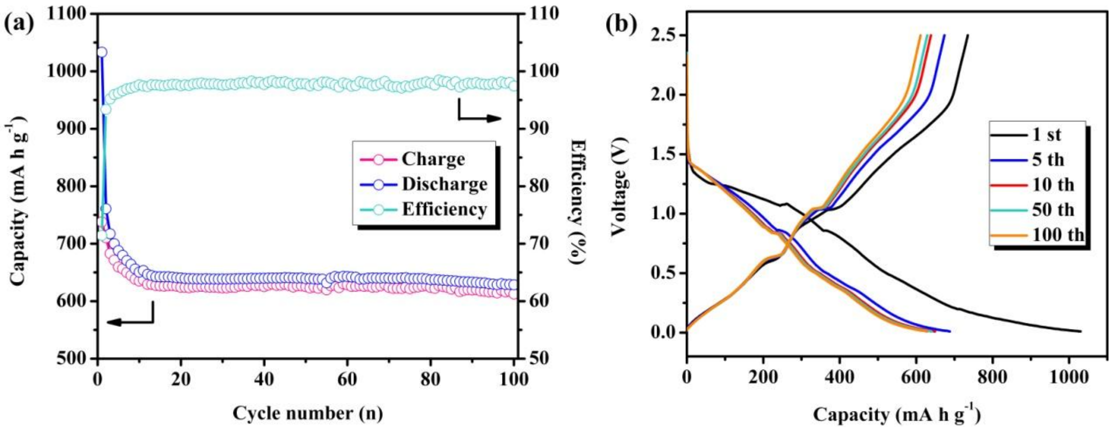

Figure. S12 Li-storage performances of I-S@MWCNTs electrodes: (a) Discharge/charge curves at different cycles at 200 mA g^{-1}; (b) Cycling performance at 200 mA g^{-1} between voltage range of 0.01 – 2.5 V.

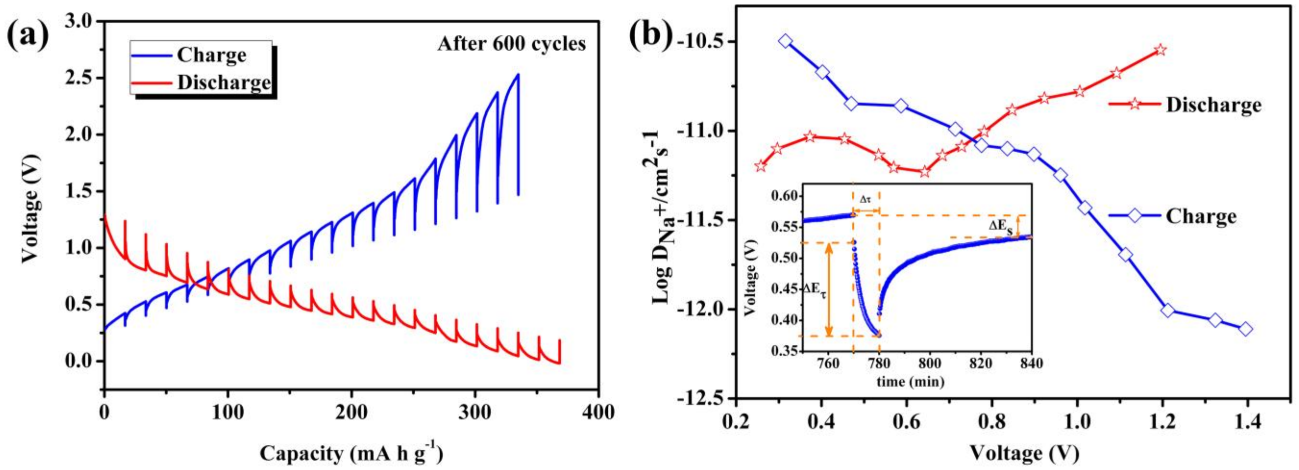

Figure. S13 (a) Galvanostatic intermittent titration technique (GITT) curves of the I-S@MWCNTs electrode after 600 cycles; the current pulse of 100 mA g^{-1} lasted for 30 min and the relaxation time was for 30 min; (b) The corresponding chemical diffusion coefficients of Na^+ ions during the discharge-charge process, inset is detailed voltage response during a single current pulse.

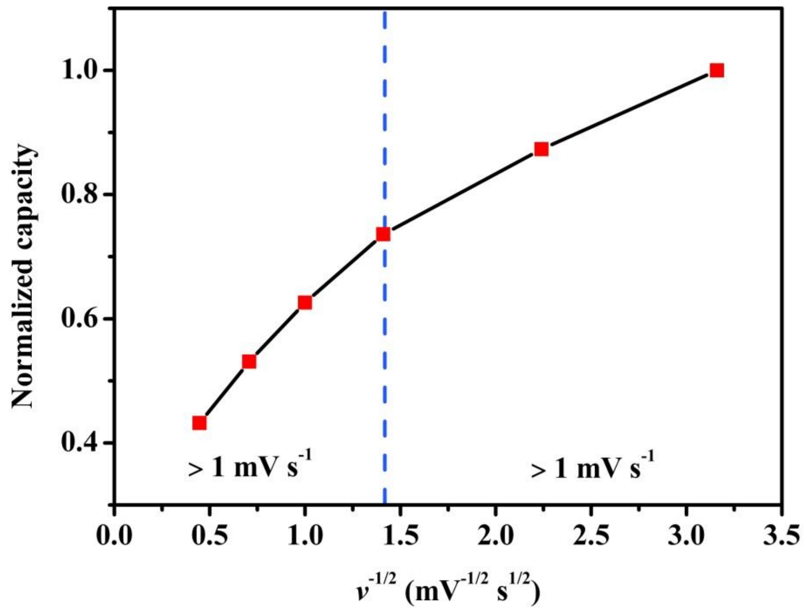

Figure. S14 Capacity versus (sweep rate)$^{-1/2}$ of I-S@MWCNTs electrode calculated from the CV data measured at different scan rates.

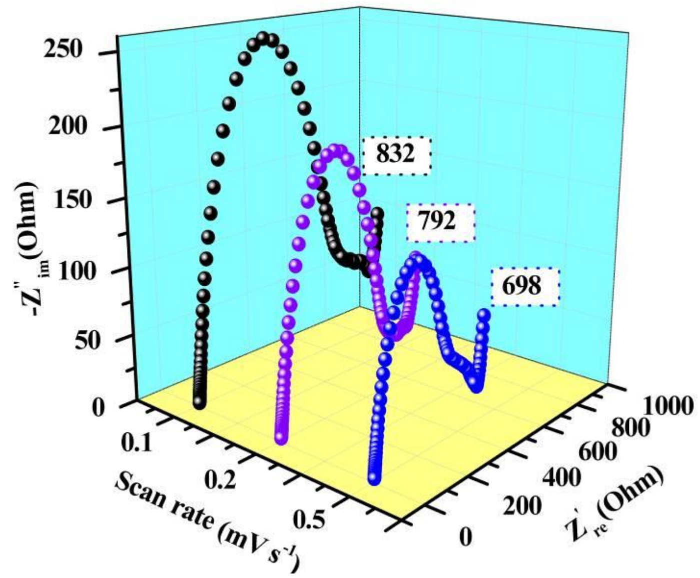

Figure. 15 EIS of In_2S_3 electrode after different scan rates.

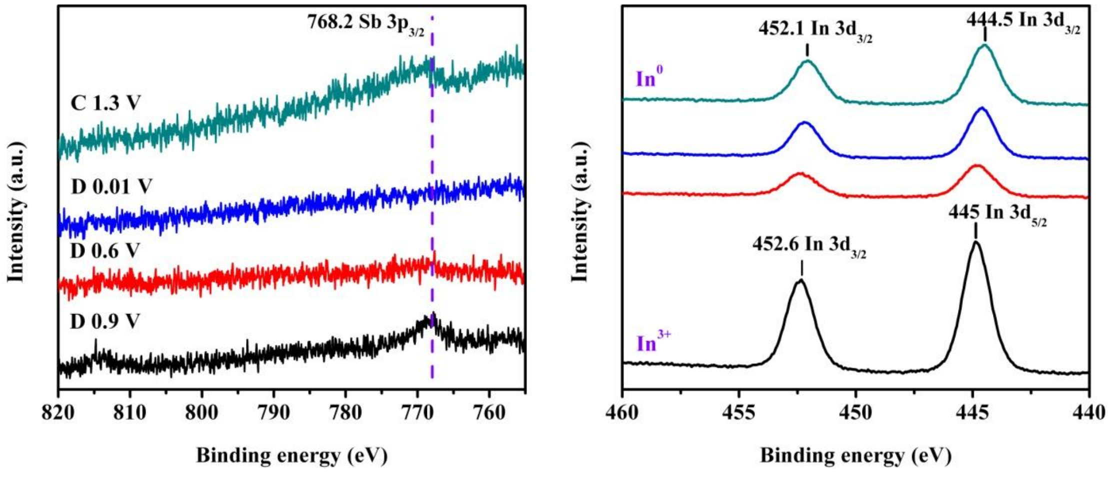

Figure. S16 *Ex-situ* XPS spectra of In 3d and Sb 3p in I-S electrode measured at D0.9 V, D0.6 V, D0.01 V and C1.3 V, respectively.

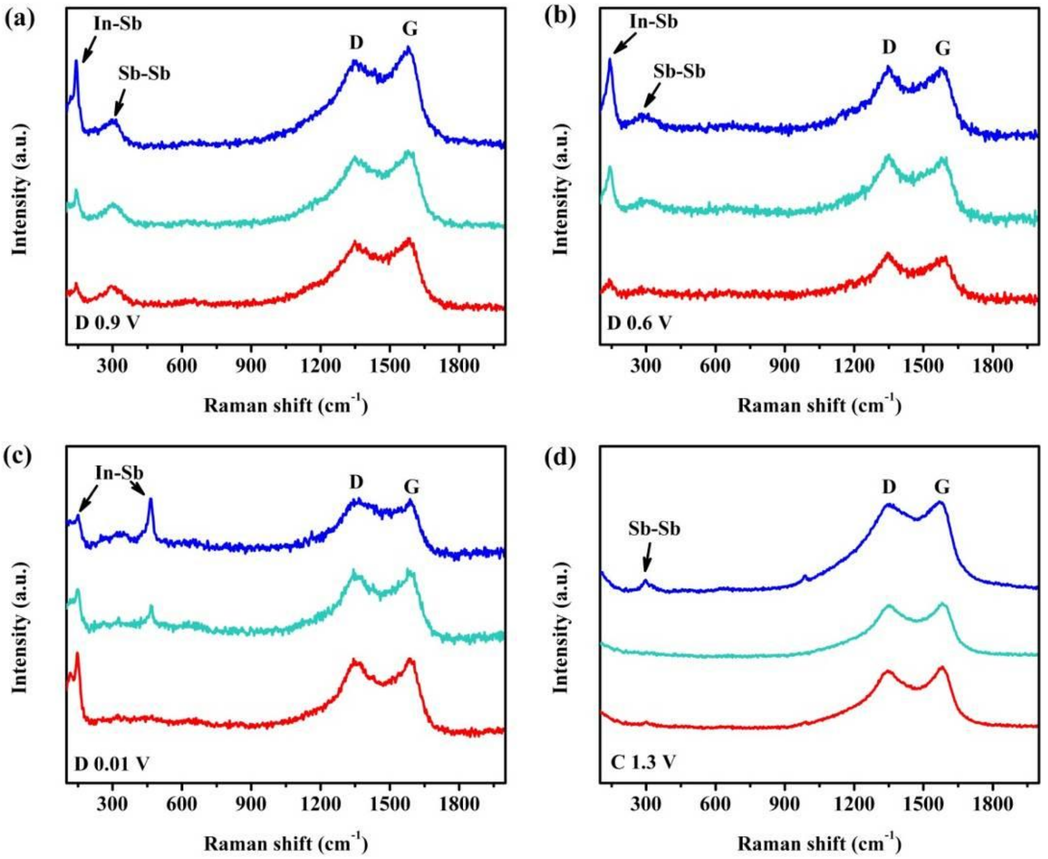

Figure. S17 *Ex-situ* Raman spectra of I-S electrodes at different charging/discharging states: (a) discharge to 0.9 V; (b) discharge to 0.6 V; (c) discharge to 0.01 V and (d) charge to 1.3 V, respectively. The red, green and blue curves correspond to the regions with the similar colors.

Table S1 Element content of the I-S and I-S@MWCNTs sample, according to the ICP-AES and elemental analysis.

Element / Sample	In % wt.	Sb % wt.	S % wt.
I-S	20.96	48.33	27.7
I-S@MWCNTs	18.95	40.75	24.7

Table S2 Summary of the representative Sb_2S_3-based anode materials for SIBs.

Typical Sample	*Cycling performances*	*Rate performances*	*References*
Sb_2S_3@C rods	Retention of 95.7 % at current density of 100 mA g^{-1} after 100 cycles	Retention of 61.3 % capacity at 100 mA g^{-1}	1
Amorphous Sb_2S_3@graphite	Retention of 89.5 % at current density of 1 A g^{-1} after 100 cycles	Retention of 73 % capacity at 10 A g^{-1}	2
Flower-like Sb_2S_3	Retention of 93.3 % at current density of 100 mA g^{-1} after 100 cycles	Retention of 76 % capacity at 2 A g^{-1}	3
Sb_2S_3 @MWCNTs	Retention of 67.5 % at current density of 0.5 A g^{-1} after 150 cycles	Retention of 76.7 % capacity at 1 A g^{-1}	4
Sb_2S_3@rGO	Retention of 95 % at current density of 50 mA g^{-1} after 50 cycles	Retention of 68.9 % capacity at 3 A g^{-1}	5
Sb_2S_3@SGS	Retention of 83.0 % at current density of 2 A g^{-1} after 900 cycles	Retention of 66.2 % capacity at 5 A g^{-1}	6
$Bi_{0.94}Sb_{1.06}S_3$	Retention of 76.8 % at current density of 0.5 A g^{-1} after 200 cycles	Retention of 45 % capacity at 10 A g^{-1}	7
In_2S_3@rGO	Retention of 93.3 % at current density of 200 mA g^{-1} after 60 cycles	Retention of 59.7 % capacity at 5 A g^{-1}	8
I-S@MWCNTs	Retention of 82.4 % at current density of 400 mA g^{-1} after 1000 cycles	Retention of 74.6 % capacity at 3.2 A g^{-1}	This work

Table S3 Calculation results of single point energies of $E_{Sb_2S_3}$, $E_{In_2S_3}$, $E_{I\text{-}S}$, $E_{Na\text{-}Sb_2S_3}$, $E_{Na\text{-}In_2S_3}$, $E_{Na\text{-}I\text{-}S}$ and E_{Na} per unit cell.

$E_{Sb_2S_3}$	$E_{In_2S_3}$	$E_{I\text{-}S}$	$E_{Na\text{-}Sb_2S_3}$	$E_{Na\text{-}In_2S_3}$	$E_{Na\text{-}I\text{-}S}$	E_{Na}
-420.7887	-1831.823	-1978.764	-1723.3122	-3134.4039	-3281.4478	-1306.5161

References:

[1] H. Hou, M. Jing, Z. Huang, Y. Yang, Y. Zhang, J. Chen, Z. Wu, X. Ji, *ACS Appl. Mater. Interfaces* **2015**, *7*, 19362.

[2] Y. Zhao, A. Manthiram, *Chem. Comm.* **2015**, *51*, 13205.

[3] Y. Zhu, P. Nie, L. Shen, S. Dong, Q. Sheng, H. Li, H. Luo, X. Zhang, *Nanoscale* **2015**, *7*, 3309.

[4] J. Li, D. Yan, X. Zhang, S. Hou, D. Li, T. Lu, Y. Yao, L. Pan, *Electrochim. Acta.* **2017**, *228*, 436.

[5] D.Y.W. Yu, P.V. Prikhodchenko, C.W. Mason, S.K. Batabyal, J. Gun, S. Sladkevich, A.G. Medvedev, O. Lev, *Nat. Commun.* **2013**, *4*, 2922.

[6] X. Xiong, G. Wang, Y. Lin, Y. Wang, X. Ou, F. Zheng, C. Yang, J. Wang, M. Liu, *ACS Nano* **2016**, *10*, 10953.

[7] Y. Zhao, A. Manthiram, *Chem. Mater.* **2015**, *27*, 6139.

[8] X. Wang, J. Hwang, S.T. Myung, J. Hassoun, Y.K. Sun, *ACS Appl. Mater. Interfaces* **2017**, *9*, 23723.
